# Astrovirus-induced epithelial-mesenchymal transition via activated TGF-β increases viral replication

**DOI:** 10.1371/journal.ppat.1009716

**Published:** 2022-04-22

**Authors:** Virginia Hargest, Theresa Bub, Geoffrey Neale, Stacey Schultz-Cherry

**Affiliations:** 1 Department of Infectious Diseases, St. Jude Children’s Research Hospital, Memphis, Tennessee, United States of America; 2 Integrated Program of Biomedical Sciences, University of Tennessee Health Science Center, Memphis, Tennessee, United States of America; 3 Hartwell Center for Bioinformatics and Biotechnology, St. Jude Children’s Research Hospital, Memphis, Tennessee, United States of America; Yale University School of Medicine, UNITED STATES

## Abstract

Human astroviruses (HAstV), positive sense single-stranded RNA viruses, are one of the leading causes of diarrhea worldwide. Despite their high prevalence, the cellular mechanisms of astrovirus pathogenesis remain ill-defined. Previous studies showed HAstV increased epithelial barrier permeability by causing a re-localization of the tight junction protein, occludin. In these studies, we demonstrate that HAstV replication induces epithelial-mesenchymal transition (EMT), by upregulating the transcription of EMT-related genes within 8 hours post-infection (hpi), followed by the loss of cell-cell contacts and disruption of polarity by 24 hpi. While multiple classical HAstV serotypes, including clinical isolates, induce EMT, the non-classical genotype HAstV-VA1 and two strains of reovirus are incapable of inducing EMT. Unlike the re-localization of tight junction proteins, HAstV-induced EMT requires productive replication and is dependent transforming growth factor-β (TGF-β) activity. Finally, inhibiting TGF-β signaling and EMT reduces viral replication, highlighting its importance in the viral life cycle. This finding puts classical strains of HAstV-1 in an exclusive group of non-oncogenic viruses triggering EMT.

## Author summary

While human astroviruses (HAstV) were discovered nearly 45 years ago, these small positive-sense RNA viruses remain critically understudied, leaving us with limited knowledge of its pathogenesis. This study fills the gap in knowledge demonstrating that HAstV are among a select group of viruses that induce epithelial-mesenchymal transition (EMT). Here we demonstrate that HAstV-1 replication, along with TGF-β signaling, induces transcriptional and phenotypic changes in intestinal epithelial cells associated with EMT. Additionally, we show inducing EMT is not common to enteric RNA viruses, as reovirus strains were found to be incapable of inducing EMT. Unlike the viruses that are known to induce EMT, astrovirus infection is not associated with cancer. We hypothesize that our findings will extend beyond astrovirus and may shed light on novel ways viruses can circumvent the barriers meant to protect against them.

## Introduction

Epithelial and mesenchymal cells share inherent plasticity that allows for switching between the two cell states through a biological process known as epithelial-mesenchymal transition (EMT). EMT is essential in the development, differentiation, and repair of tissues and organs; however, EMT can negatively contribute to organ fibrosis and the initiation of cancerous metastases. EMT has been shown to induce stem cell properties [1,2], prevent apoptosis and senescence [3–5], and contribute to immunosuppression [6]. The complex process of EMT involves extensive reprogramming of gene expression, which can be regulated by numerous signaling pathways [7]. Activation of these signaling pathways ultimately results in the upregulation of the transcription factors including Snail1/2, Twist, and ZEB1/2 [7]. These transcription factors negatively regulate epithelial markers such as occludin, claudins, and E-cadherin, while positively regulating mesenchymal genes like N-cadherin, fibronectin, and vimentin [8]. This allows for the hallmark phenotypic changes associated with EMT like the disassembly of the epithelial cell-cell junctions, the loss of apical-basal polarity, and the formation of lamellipodia or filopodia to enable migration [9].

Epithelial cells that line the intestinal lumen function as a barrier that absorbs nutrients and electrolytes while restricting entry of harmful substances or pathogens [10]. Breaches in this barrier by death of the epithelial cells or disruption of cellular junctions through non-cytopathic mechanisms are associated with gastrointestinal diseases including irritable bowel syndrome, Crohn’s disease, and colitis [11]. Along with these diseases, enteric viruses are known to compromise the gastrointestinal barrier. Human astroviruses (HAstV), small, non-enveloped positive-sense single-stranded RNA viruses, have been shown to predominantly infect differentiated epithelial cells at the tips of the intestinal villi [12,13]. We have demonstrated that astrovirus disrupts the intestinal barrier through a novel mechanism independent of cellular damage or induction of the host inflammatory response [14–16]. Instead, astroviruses increase barrier permeability by inducing the re-localization of the tight junction protein occludin [15]. The re-localization of occludin by astrovirus does not require productive infection; the viral capsid protein alone is sufficient to cause disruption *in vivo* and *in vitro* [15,16]. Because loss of cell junctions is a phenotypic hallmark of EMT, we hypothesized that HAstV may serve as a viral trigger of EMT.

Indeed, several viruses are known to induce EMT including hepatitis B virus (HBV) [17], hepatitis C virus (HCV) [18], human papilloma virus (HPV) [19], Epstein-Barr virus (EBV) [20], and cytomegalovirus (CMV) [21,22]. These viruses, unlike HAstV, are oncogenic, and EMT induction leads to metastases, hepatocellular carcinoma, cervical carcinomas, and lymphoma among other diseases. Here, we demonstrate that HAstV is a non-oncogenic virus that also drives EMT. HAstV-induced EMT begins with an upregulation of EMT related genes and transcription factors at 8 hours post-infection (hpi). This is followed by a loss of epithelial cell-specific genes and proteins and gain of mesenchymal proteins like vimentin by 24 hpi. It is also accompanied by a loss of cellular polarity. While multiple classical HAstV serotypes are capable of inducing EMT, other enteric viruses and even non-classical HAstV genotypes do not trigger EMT. We demonstrate that HAstV activates the well-established inducer of EMT, TGF-β, and HAstV-induced EMT is dependent on this TGF-β signaling as well as productive viral replication. In fact, when TGF-β signaling is inhibited viral replication is significantly reduced. The studies described here are amongst the first to demonstrate that a non-oncogenic virus drives EMT and the importance of EMT in the viral life cycle.

## Results

### HAstV infection leads to EMT-associated gene modulation

**Fig 1:**
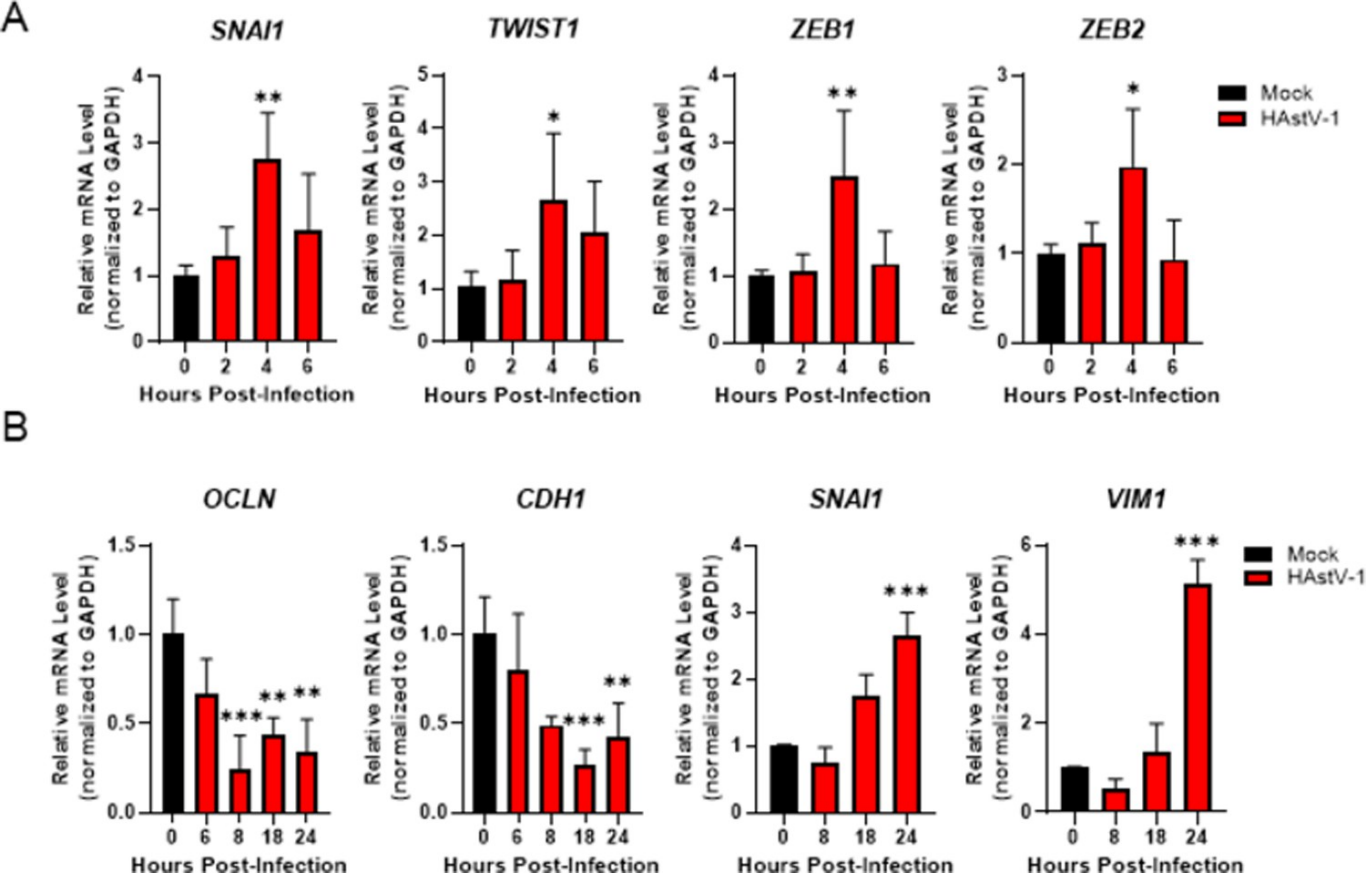
HAstV-1 infection leads to a decrease in epithelial markers while increasing mesenchymal markers. (A) Early in infection (2–6 hpi) EMT transcription factors (*SNAI1*, *TWIST1*, *ZEB1* and *ZEB2*) are upregulated causing (B) epithelial genes (*OCLN* and *CDH1*) to be down regulated and mesenchymal genes (*SNAI1* and *VIM*) to be upregulated Later (6–24 hpi) HAstV-1 infection. Error bars indicate standard deviations from two independent experiments performed in triplicate, and asterisks show statistical significance as measured by ordinary one-way ANOVA followed by Dunnett’s multiple comparisons test as follows: *, P < 0.05; **, P < 0.01; ***, P < 0.001.

We have previously demonstrated that HAstV-1 leads to reorganization of occludin and the actin cytoskeleton without causing cell death [15]. This observation led us to hypothesize that HAstV infection induces EMT. Since EMT is a transcriptionally regulated process, we sought to determine if EMT-associated genes were modulated during HAstV-1 infection by first performing microarray analysis. Gene set enrichment analysis (GSEA) of the microarray data demonstrated that several pathways were significantly upregulated in HAstV-infected versus uninfected Caco-2 cells at 24 hpi, including the EMT pathway (S1 Fig). To investigate this further, we performed more targeted quantitative analysis by examining mRNA levels of EMT associated genes throughout infection by multiplexed qRT-PCR using Qiagen’s RT^2^ Profiler system (S2 Fig). Cellular pathways associated with the induction of EMT were upregulated at 8 hpi including, Wnt, TGF-β, and Notch, as were specific transcription factors known to drive EMT including Snail, ZEB1/2, and Twist. We validated the RT^2^ Profiler findings by quantitating mRNA levels of EMT-related transcription factors as well as E-cadherin (*CDH1*), occludin (*OCLN*), Snail (*SNAI1*), and vimentin (*VIM*) by qRT-PCR. We observed a modest upregulation of the EMT-related transcription factors *SNAI1*, *TWIST1*, *ZEB1*, and *ZEB2* around 4 hpi (Fig 1A), indicating transcriptional changes may be taking place very early in the replication cycle. The activation of these transcription factors was followed by the downregulation of epithelial genes, *CDH1* and *OCLN*, as early as 8 hpi and the upregulation of mesenchymal gene *VIM* by 24 hpi (Fig 1B). The upregulation of *SNAI1* was not sustained throughout infection but instead may be regulated in a biphasic manner as another increase in mRNA level was observed at 18 to 24 hpi (Fig 1B).

### HAstV infection leads to a time-dependent reorganization and decrease in junctional protein levels

**Fig 2:**
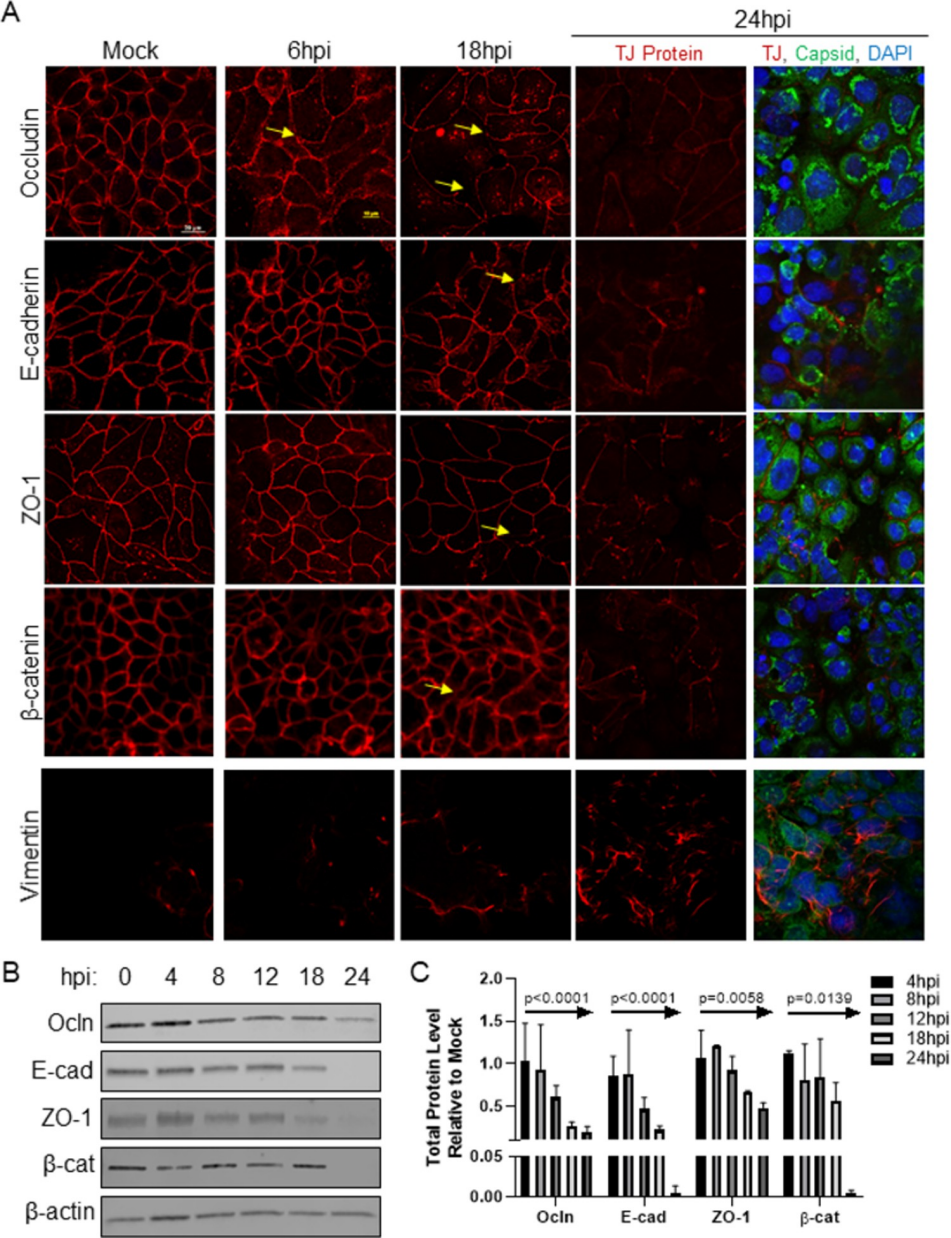
HAstV-1 infection leads to a time-dependent decrease in junctional protein levels. (A) Caco-2 monolayers on grown coverslips, infected with HAstV-1 (MOI of 10) or mock infected. Cells were fixed at 6, 18, and 24 hpi in 4% paraformaldehyde and then stained for the indicated junctional proteins alone or with astrovirus capsid (green) and DAPI (blue). Arrows indicate areas of junctional disruption. Images are representative of at least three independent experiments. (B) Expression of epithelial markers, occludin (Ocln), E-cadherin (E-cad), ZO-1, and β-catenin (β-cat), were quantified by immunoblot of HAstV-1 or mock infected Caco-2 cell lysates. (C) Bands were then quantified by densitometry and normalized to β-actin then compared to mock-infection. Error bars indicate standard deviations three independent experiments performed in duplicate; p-value as measured by Ordinary One-way ANOVA followed by a test for trend is indicated for each protein.

Given the transcriptional changes, we asked if junctional protein expression was also disrupted during HAstV infection. To examine this, Caco-2 grown on glass coverslips were infected with HAstV-1 and stained for tight junction proteins, occludin and zonula occludens-1 (ZO-1), and adherens junction proteins, E-cadherin and β-catenin, at 6, 12, 18, and 24 hpi. Mock-infected cells showed normal cell junction morphology, with a cobblestone-like staining pattern as protein localization was restricted to the cell periphery. However, HAstV-infected cells had disrupted junctional proteins. The disruption was as early as 6 hpi with occludin beginning to re-localize away from the cell periphery (Fig 2A). Re-localization of occludin was followed by ZO-1 moving from the cell membrane around 18 hpi. Despite the significant change in the distribution of the junctional proteins and consistent with previous findings [14–16,23], our observation was not caused by any significant cell death (S3 Fig). The most striking finding was the reorganization of E-cadherin, a key marker of epithelial cells [24], by 18 hpi. E-cadherin is crucial in the establishment and maintenance of the cellular junction complex as a whole [25,26], and aberrant expression of E-cadherin is a hallmark of epithelial dysregulation [7]. Additionally, we observed an increase in vimentin staining correlating to the increase in vimentin mRNA. Not only did we observe cellular junction reorganization, but the overall proteins levels were also decreased. Over the course of 24 hours, expression of the junctional proteins occludin (p<0.0001), E-cadherin (p<0.0001), ZO-1 (p = 0.0058), and β-catenin (p = 0.0139) were all significantly decreased relative to mock-infected cells (Fig 2B and 2C), indicating the observed transcriptional changes during HAstV infection translated to a decrease in junctional protein expression.

### HAstV-induced EMT disrupts cellular polarity

**Fig 3:**
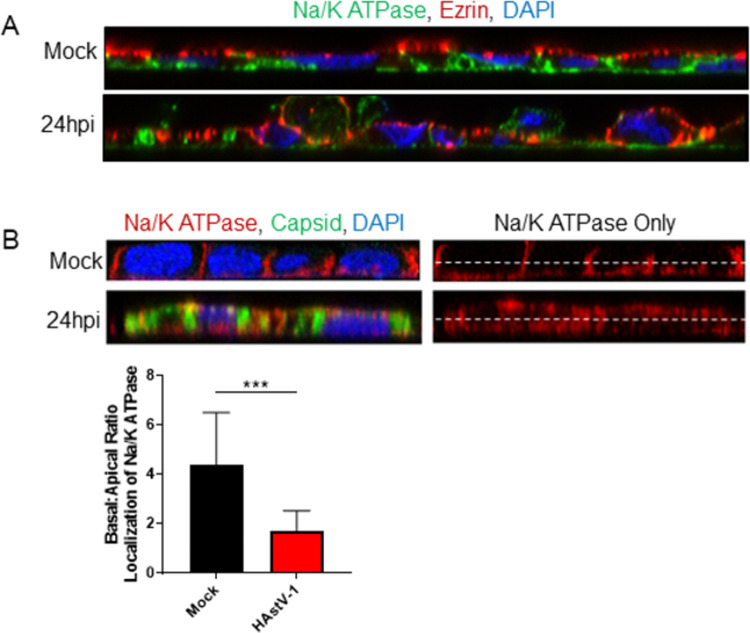
HAstV-1 infection leads to a disruption of cellular polarity. (A) Caco-2 infected with HAstV-1 or mock infected (as indicated). Cells were fixed at 24 hpi in 100% ice-cold methanol and then stained for ezrin (red), sodium-potassium ATPase (green), and DAPI (blue). Images are representative of at least three independent experiments. (B) Basal or apical localization of sodium-potassium ATPase was determined by measuring intensity above and below cell midline of HAstV- or mock-infected cells using ImageJ. Error bars indicate standard deviations of three independent experiments performed in triplicate, and asterisks show statistical significance as measured by the two-tailed Student *t* test as follows: ***, P < 0.001.

Cellular junctions act as a physical barrier that prevent the movement of lipids and membrane proteins from migrating between the apical and basolateral cell membranes [27].When cellular junctions are disassembled, proteins that were once localized to the basolateral membrane freely migrate to the apical side causing a loss of cellular polarity [28]. To determine if cellular polarity was disrupted during HAstV-1 infection, we stained for ezrin, a cytoplasmic linker between the apical membrane and the actin cytoskeleton [29], and sodium-potassium ATPase (Na/K-ATPase), a transporter localized to the basolateral membrane [30]. In mock-infected cells ezrin was distinctly localized to the apical side and Na/K-ATPase to the basolateral with very little overlap (Fig 3A). However, by 24 hpi there was less organized arrangement for both proteins. To quantitate the disruption of polarity, we measured the amount of Na/K-ATPase at apical membrane. At 24 hpi, there was significantly more Na/K-ATPase located at the apical membrane than in mock-infected cells (Fig 3B). We also noticed that cells appeared to lift or be extruded from the cell monolayer (Fig 3A; bottom panel). The transcriptional reprogramming, disassembly of epithelial cell-cell contacts, especially the disruption and decreased production of E-cadherin, and disruption of cellular polarity all indicated that during HAstV-1 infection cells were undergoing EMT.

### The induction of EMT is unique to HAstV

**Fig 4:**
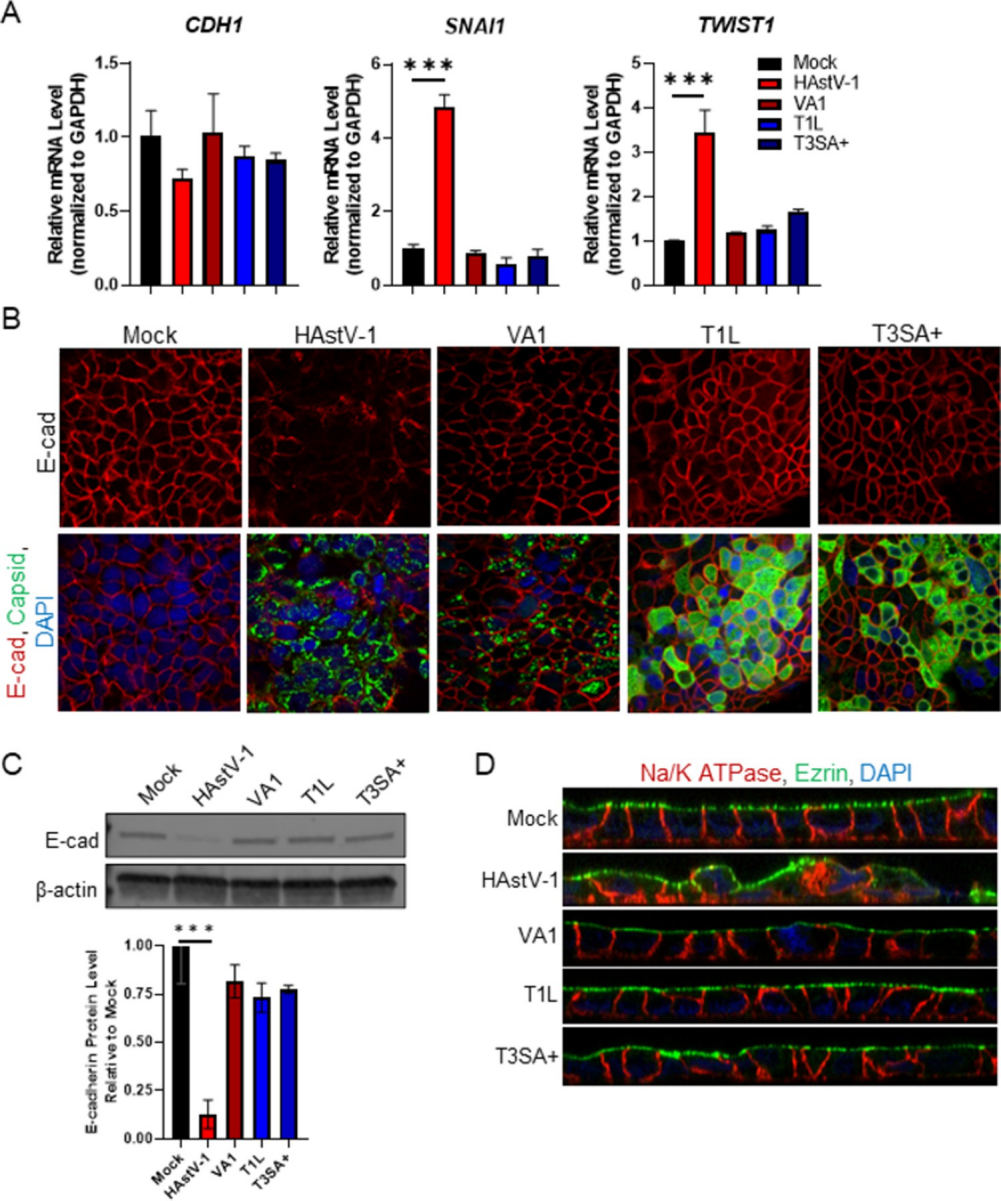
Other enteric viruses do not induce EMT in Caco-2 cells. (A) Caco-2 monolayers grown on permeable supports were infected with HAstV-1, HAstV-VA1, T1L, T3SA+ (MOI of 10) or mock-infected and TER (transepithelial electrical resistance) was measured from 0–24 hpi. (B) RNA extracted at 24 hpi from Caco-2 cells infected with HAstV-1, HAstV-VA1, T1L, T3SA+, or mock infected, show only HAstV-1 modulate *CDH1*, *SNAI1*, and *TWIST1*. (C) Infected cells were fixed at 24 hpi and stained for E-cadherin (red), viral capsid (green), and DAPI (blue). (D) Expression of E-cadherin was quantified by immunoblot of HAstV-1, HAstV-VA1, T1L, T3SA+ infected (MOI of 10) or mock infected Caco-2 cell lysates. Bands were then quantified by densitometry and normalized to β-actin then compared to mock-infection. (E) Na/K ATPase (red) and ezrin (green) localization is disrupted in cells infected with HAstV-1 compared to HAstV-VA1, T1L, T3SA+, and mock infected cells. All error bars indicate standard deviations of two independent experiments performed in triplicate, and asterisks show statistical significance as measured by ordinary one-way ANOVA followed by Dunnett’s multiple comparisons test as follows: *, P < 0.05; **, P < 0.01; ***, P < 0.001. All images are representative of two independent experiments.

With our phenotype characterized, we next determined the breadth of HAstV strains capable of inducing EMT. Caco-2 cells were infected with three classical HAstVs isolated from patient samples, SJ054.225 (HAstV-1), SJ60.212 (HAstV-8), and SJ177.110 (HAstV-2), as well as lab adapted classical serotypes HAstV-8 and HAstV-2. All clinical isolates and lab-adapted serotypes disrupted the localization (S4A Fig) and expression (S4B Fig) of E-cadherin. Additionally, infection with these viruses disrupted polarity as demonstrated by re-localization of both ezrin and Na/K ATPase (S4C Fig). In contrast, the non-classical HAstV-VA1 genotype, which shares only 33% homology with HAstV-1 [31], failed to downregulate *CDH1* or upregulate *SNAI1* or *TWIST1* (Fig 4A) despite productively replicating in the Caco-2 cells (Fig 4B). The lack of EMT was not unique to HAstV-VA1. Reovirus stains T1L and T3SA+, while also productively replicated in Caco-2 cells, failed to disrupt E-cadherin protein localization (Fig 4B) or expression (Fig 4C). Finally, neither reovirus strain nor HAstV-VA1 were able to disrupt cellular polarity upon infection (Fig 4D). This shows classical HAstV is distinct among other enteric RNA viruses in the ability to induce EMT.

### HAstV-induced EMT is dependent on viral replication

**Fig 5:**
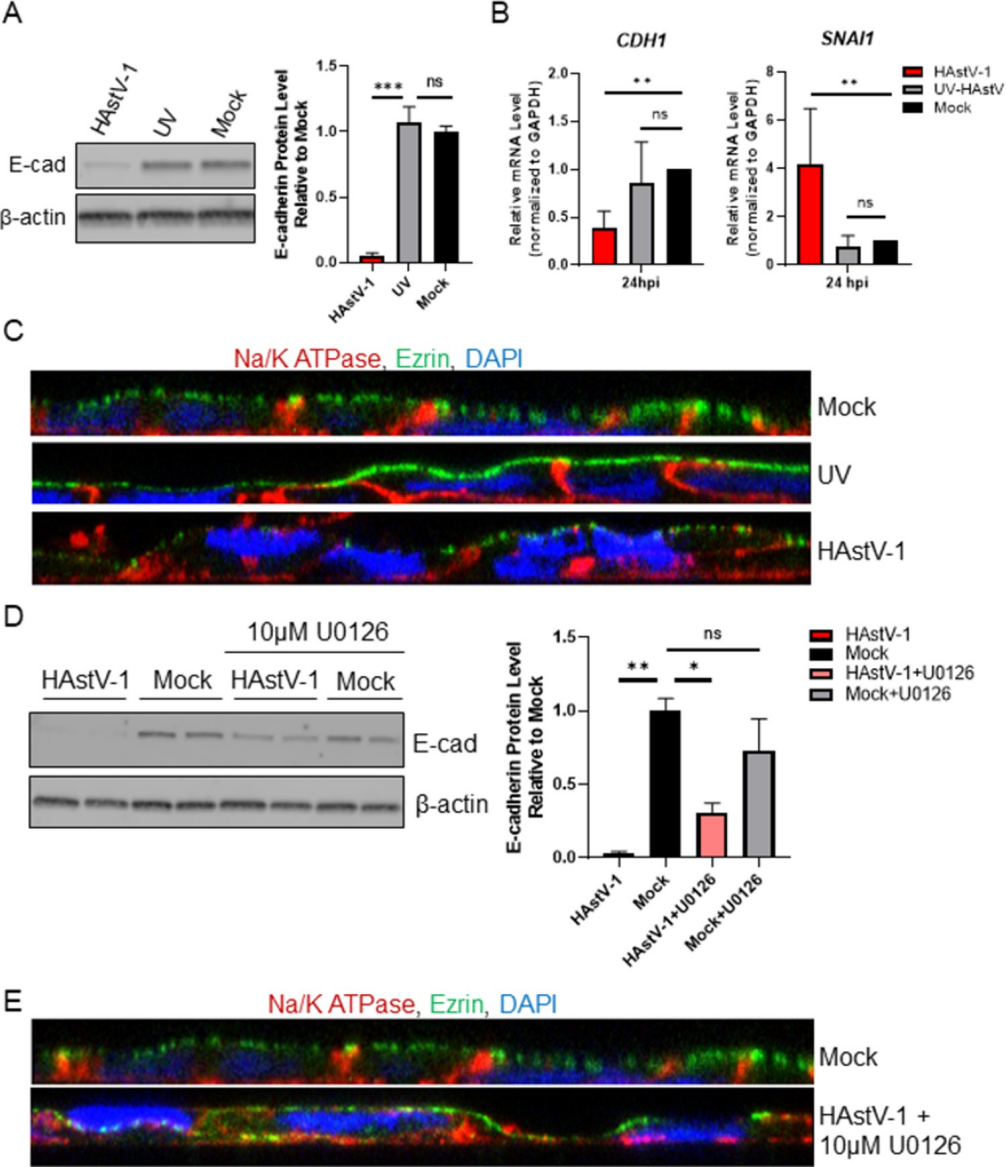
Replication is required for HAstV-1 induced EMT. (A) Expression of E-cadherin was quantified by immunoblot of HAstV-1, UV-inactivated HAstV-1, or mock infected Caco-2 cell lysates. Bands were then quantified by densitometry and normalized to β-actin then compared to mock-infection. (B) RNA extracted at 24 hpi from Caco-2 cells infected with HAstV-1, UV-inactivated HAstV-1, or mock infected show UV-inactivated virus does not modulate *CDH1* or *SNAI1* regulation as active HAstV-1 does. (C) Na/K ATPase (red) and ezrin (green) localization in Caco-2 cells inoculated with UV-inactivated HAstV-1 shows no difference to mock infected cells. (D) Expression of E-cadherin was quantified by immunoblot of HAstV-1, HAstV-1 + 10μM U0126, or mock infected Caco-2 cell lysates. Bands were then quantified by densitometry and normalized to β-actin then compared to mock-infection. (E) Na/K ATPase (red) and ezrin (green) localization in Caco-2 cells infected with HAstV-1 in the presence of 10μM U0126 and mock infected cells. All error bars indicate standard deviations of three independent experiments performed in triplicate, and asterisks show statistical significance as measured by ordinary one-way ANOVA followed by Dunnett’s multiple comparisons test as follows: *, P < 0.05; **, P < 0.01; ***, P < 0.001.

Our previous research has shown that the astrovirus capsid protein alone can cause significant barrier permeability [15,16], we examined if capsid protein alone is sufficient to induce EMT. To test this, we inoculated Caco-2 cells with UV-inactivated virus and assessed the hallmarks of EMT. UV-inactivated virus did not increase *SNAI1* mRNA, decrease *CDH1* leading to reduced E-cadherin expression, or disrupt cellular polarity (Fig 5A–5C). Further, inhibition of ERK1/2, which is critical for HAstV replication [23], with U0126, rescued more than 27% of E-cadherin expression and cellular polarity (Fig 5D and 5E). Additionally, to establish that our phenotype is not an artifact of the Caco-2 cell line, we tested HT29 cells for our hallmarks of HAstV-induced EMT. While we did observe a decrease in E-cadherin transcription and expression at 24 hpi as well as a slight increase in *SNAI1* transcription (S5A–S5C Fig), it was to a much lesser extent than in Caco-2 cells. However, HT29 cells are significantly less permissive to HAstV-1 infection. When infected at a MOI of 10, approximately 90% of the cell monolayer is infected by 24 hpi, while HT29 monolayers exhibit roughly 30% infection (S5A Fig). These studies suggest that productive replication is required for the induction of EMT.

### Inhibition of TGF-β signaling during HAstV-1 infection inhibits HAstV-induced EMT and reduces viral replication

**Fig 6:**
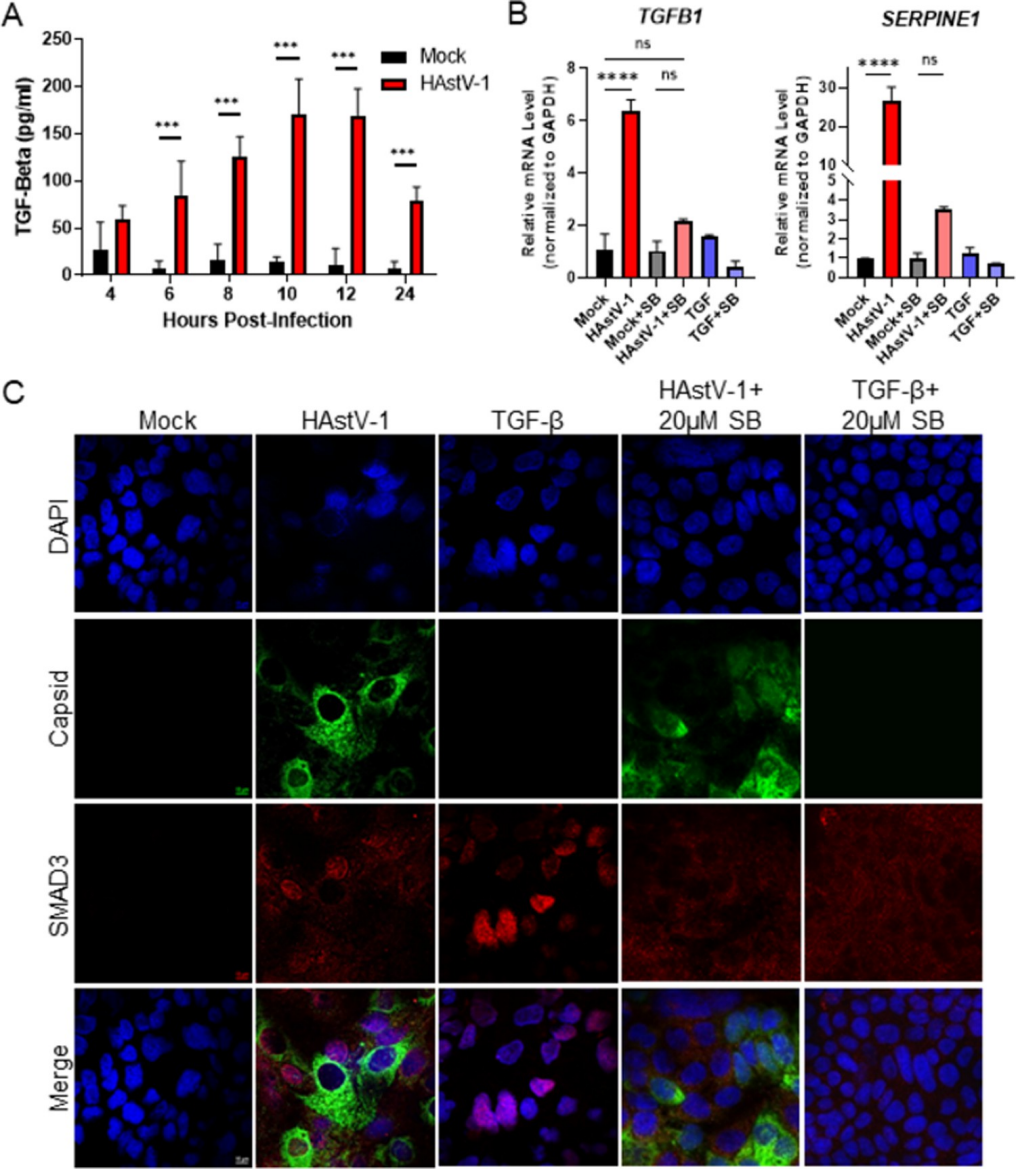
TGF-β activity increases during HAstV-1 infection. (A) Supernatants were collected from Caco-2 infected with HAstV-1 or mock infected from 4 to 24 hpi. Supernatants were then assayed for active TGF-β using the PAI assay as described previously [34]. Error bars indicate standard deviations from two independent experiments performed in duplicate, and asterisks show statistical significance as measured by two-way ANOVA followed by Sidak’s multiple comparisons test as follows: *, P < 0.05; **, P < 0.01; ***, P < 0.001. (B) *TGFB1* and *SERPINE1*, which is specifically activated by TGF-β, mRNA levels were measured in mock infected, HAstV-1 infected (MOI 5), or TGF-β (20ng/ml) treated Caco-2 cells at 24 hpi. Error bars indicate standard deviations from two independent experiments performed in duplicate, and asterisks show statistical significance as measured by ordinary one-way ANOVA followed by Tukey’s multiple comparisons test as follows: ****, P < 0.0001. (C) Caco-2 cells mock infected, HAstV-1 infected, or treated with TGF-β with and without 20μM SB431542 treatment were stained for SMAD3 (red), astrovirus capsid protein (green) and DAPI (blue). Images are representative of two independent experiments.

When examining the upregulation of mesenchymal genes, we observed that TGF-β mRNA was increased at both 8 and 24 hpi (S2 Fig). Since TGF-β is the classical activator of EMT [7,32,33], we asked whether this increase in mRNA translated to an increase in TGF-β activity. To measure active TGF-β levels, we utilized a specific biological reporter assay where mink lung epithelial cells (Mv1Lu) stably express the PAI promoter upstream of luciferase [34]. Supernatants collected from HAstV-1 infected Caco-2 cells between 4 and 24 hpi were added to the Mv1Lu-PAI cells and TGF-β activity was quantitated. Supernatants from HAstV-1- infected cells contained significantly more active TGF-β compared to mock-infected cells beginning at 6 hpi and peaking at 12 hpi (Fig 6A). We also observed an increase in SMAD3 nuclear localization in HAstV-1-infected cells comparable to cells treated with TGF-β alone and upregulation of *SERPINE1* mRNA, which is specifically activated by TGF-β, mRNA at 24 hpi (Fig 6B and 6C). These studies demonstrate that astrovirus infection leads to an increase in biologically active TGF-β.

**Fig 7:**
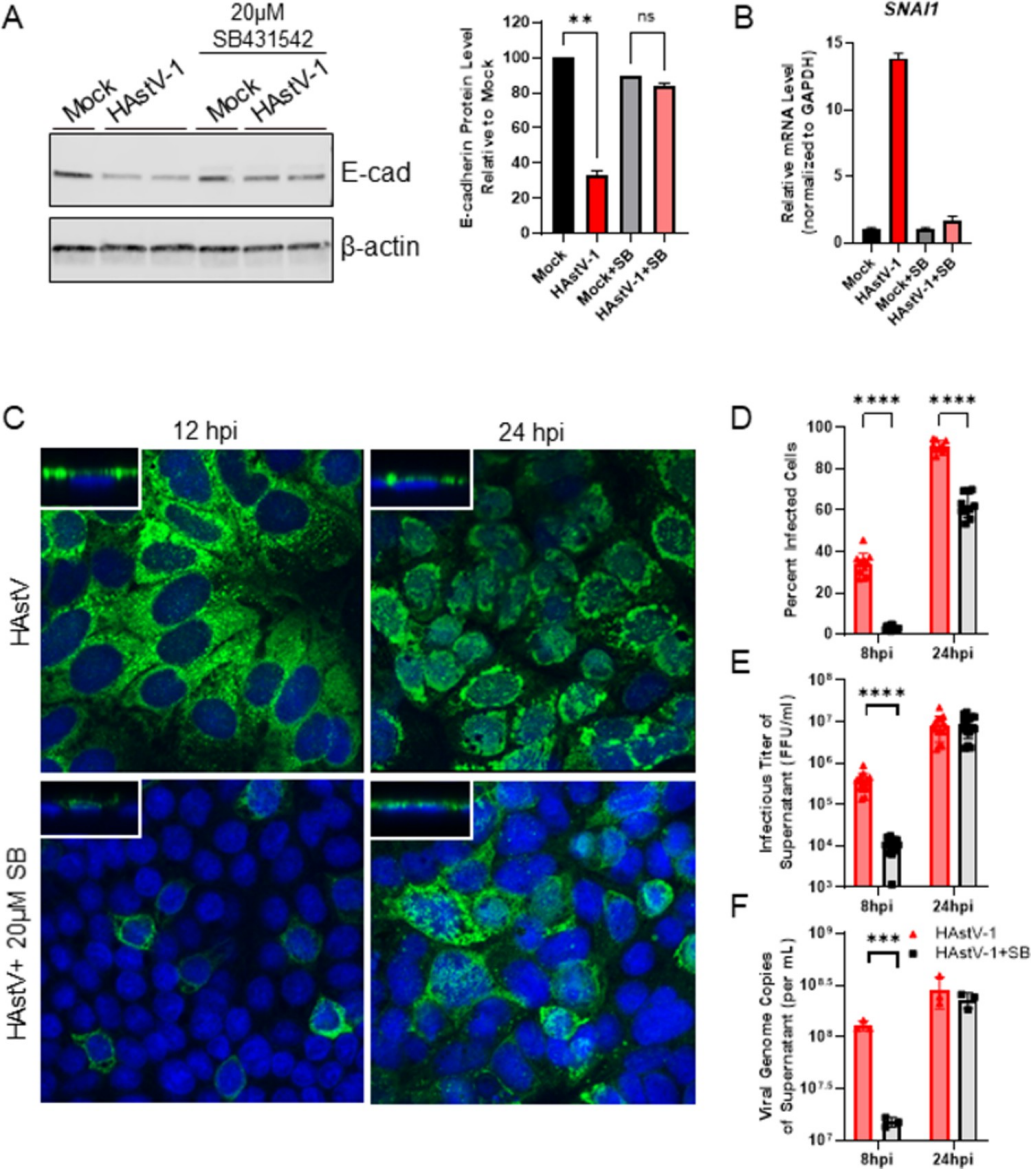
Inhibition of TGF-β signaling with SB431542 inhibits HAstV-1 induced EMT and reduces viral replication. (A) Expression of epithelial marker, E-cadherin, was quantified by immunoblot of HAstV-1 or mock infected Caco-2 cell lysates with or without 20μM SB431542. Bands were then quantified by densitometry and normalized to β-actin then compared to mock-infection. Error bars indicate standard deviations of two independent experiments performed in duplicate, and asterisks show statistical significance as measured by ordinary one-way ANOVA followed by Tukey’s multiple comparisons test as follows: **, P < 0.01. (B) RNA extracted at 24 hpi from Caco-2 cells infected with HAstV-1 or mock infected with or without 20μM SB431542 shows inhibiting TGF-β abolished *SNAI1* up-regulation in HAstV-induced EMT. (C) HAstV-1 infected Caco-2 cells stained for capsid protein (green) and DAPI (blue) at 12 and 24 hpi reveal SB431542 treatment reduces the percent of infected cells. Additionally, SB431542 may alter viral egress, as more capsid staining is localized directly above the nucleus in treated versus non-treated cells (z-stack insets). Images are representative of two independent experiments. (D) The percent of HAstV-1 infected cells with and without SB431542 treatment were quantified at 8 and 24 hpi, supernatant titers quantified by FFU and genome copy number. Error bars indicate standard deviations of two independent experiments performed in triplicate, and asterisks show statistical significance as measured by two-way ANOVA followed by Sidak’s multiple comparisons test as follows: ****, P < 0.0001. The titer of supernatant of HAstV-1 infected cells with and without SB431542 treatment were quantified at 8 and 24 hpi by FFU (E) and qPCR for viral genome copies (F). Error bars indicate standard deviations of two independent experiments performed in triplicate, and asterisks show statistical significance as measured by unpaired t-test as follows: ***, P < 0.001; ****, P < 0.0001.

To determine if HAstV-induced EMT was dependent on this active TGF-β, TGF-β signaling was inhibited using the small molecule inhibitor SB431542 [35], which selectively inhibits the phosphorylation of the TGF-β type I receptor. Inhibiting TGF-β signaling with 20μM SB431542, abolished the upregulation of *SERPINE1* and *TGFB1* mRNA at 24 hpi and SMAD3 nuclear localization (Fig 6B and 6C). When examining our hallmarks of EMT following SB431542 treatment, we observed a restoration of E-cadherin protein expression to that of mock-infected cells (Fig 7A) and an elimination of *SNAI1* up-regulation associated with HAstV-1-induced EMT (Fig 7B). The addition of 20 ng/ml recombinant TGF-β1 alone did not induce EMT within the same time frame as HAstV. The loss of E-cadherin and disruption of polarity was not seen until 3 days following TGF-β administration (S6 Fig). This indicates that TGF-β in conjunction with HAstV-1 replication is necessary to induce EMT.

When we examined how TGF-β inhibition affected viral replication, we found that SB431542 treatment significantly reduced the percent of HAstV-1 infected cells at both 8 and 24 hpi (Fig 7C and 7D). In addition, the was a significant decrease in the infectious viral titer and genome copies at 8 hpi, possibly indicating a delay in viral release and spread from SB431542 treated cells (Fig 7E and 7F). In fact, when looking at the viral capsid staining pattern, we observed SB431542 treated cells retained capsid protein located above the nucleus where untreated cells have little to none (Fig 7C; z-stack inset). Taken together, our findings confirm HAstV-1 infection induces TGF-β dependent EMT that allows for efficient viral replication and release.

## Discussion

In these studies, we demonstrate that HAstV replication triggers EMT in Caco-2 cells. Mechanistically, HAstV-induced EMT is driven by transcriptional changes; specifically, the downregulation of *CDH1* and *OCLN* and the upregulation of the key transcriptional factor *SNAI1* early after infection. These transcriptional changes result in decreased epithelial protein levels, leading to a breakdown of the cell junctions, the loss of cellular polarity, and upregulation of mesenchymal cell-specific genes like *VIM* by 24 hpi. TGF-β activity increases during HAstV infection, and inhibition of TGF-β signaling prevents the EMT phenotype and impacts viral replication. The data we present showed that HAstV replication along with TGF-β signaling causes the transcriptional and phenotypic changes associated with EMT.

Previously published data from our lab has shown TGF-β activity is upregulated *in vivo* in the turkey poult model [14]. We had hypothesized TGF-β was important *in vivo* to create an immune suppressive microenvironment [36] and contributed to the lack of inflammation seen with astrovirus infections. Our data suggests that TGF-β is important for HAstV-induced EMT. The induction of EMT may actually account for how astrovirus can breach the intestinal barrier and cause the viremia seen in the turkey poult model [14] as well as some human cases [37–39]. Additionally, our findings indicate TGF-β signaling is required for efficient replication and release of the virus. We observed a decrease in not only the number of infected cells, but the amount of virus released in SB431542 treated cells (Fig 7D). While the percent of infected cells was unable to recover this deficit by 24 hpi, the viral titer recovered in the supernatant did (Fig 7E and 7F). This likely means inhibition of TGF-β only delays HAstV replication rather than blocking it. However, we cannot rule out that the rebound in replication is due to the half-life of SB431542. Our SMAD3 staining showed that TGF-β signaling is frequently occurring in non-infected cells in the epithelial monolayer. This phenomenon brings into question whether EMT is induced in directly infected cells or if TGF-β signaling in surrounding cells triggers EMT in reaction to HAstV infection. Future studies will investigate the activation of TGF-β during astrovirus infection and role of TGF-β signaling in bystander cells.

While multiple classical HAstV serotypes induced EMT, this was not true of all astrovirus genotypes. HAstV-VA1 failed to drive EMT despite productively replicating in Caco-2 cells. We showed that HAstV-VA1 failed to upregulate the transcription factor *SNAI1* during a 24-hour infection. The lack of *SNAI1* regulation is a key finding as we observed multiple waves of *SNAI1* upregulation with HAstV-1 infection (Fig 1), which is likely a driving force in HAstV-induced EMT. HAstV-VA1 is a non-classical HAstV strain, genetically more related to mink and ovine astroviruses than to the classical human serotypes [40–42]. Unlike classical HAstVs, HAstV-VA1 has rarely been linked to diarrhea [43,44] but has been reported in association with neurological disease [31,45]. Classical and non-classical HAstVs are genetically distinct and differ on key factors of replication and pathogenesis [46,47]. Since the novel HAstV strain was discovered just over 10 years ago, investigations into its pathogenesis are just beginning and our finding that it does not induce EMT may reveal an underlying fundamental difference between it and classical HAstVs.

The induction of EMT is also not a characteristic of other enteric RNA viruses. Reovirus, like HAstV, is a small RNA virus that can cause severe diarrhea in children [48]. Yet, two separate strains of reovirus were unable to cause the same phenotypic or transcriptional hallmarks of EMT. Attempts to examine coxsackievirus (CVB3)-induced EMT were unsuccessful due to the cytopathogenic nature of this virus. The lack of EMT induction by other enteric viruses also indicates this is not a phenomenon the Caco-2 cell line used as our *in vitro* model. Since Caco-2 cells are a carcinoma cell line, it could be suggested these cells are simply predisposed to undergo EMT. However, all viral infections were carried out under the same conditions in the Caco-2 cell line. Given the other viral stains did not induce EMT, our findings are not an artifact of the cells. EMT is a rare phenomenon triggered by only a few viruses and even less non-oncogenic viruses, making HAstV-induced EMT a truly unique finding.

Our previous research has shown the astrovirus capsid alone can increase barrier permeability and disrupt cellular junctions *in vitro* and *in vivo* [15,16]. Conversely, when HAstV-1 is UV-inactivated, preventing the virus from replicating its genome, it no longer is capable of inducing EMT. Additionally, when viral replication was suppressed using the ERK1/2 inhibitor U0126, we saw a decline in the hallmarks of EMT. The addition of U0126 to HAstV infection did not completely reverse the effects of EMT, however this was expected as U0126 does not completely inhibit HAstV replication [23]. We hypothesize that there may be a binding event that is sufficient to cause some barrier permeability and the re-localization of occludin thus allowing the capsid protein alone to cause these events. However, a more intricate signaling cascade is triggered during replication of the virus that induces the EMT phenotype. While further research is needed to determine the exact aspect of replication that is initiating EMT, our current hypothesis is the production and activity of one of the HAstV non-structural proteins is involved.

In conclusion, we demonstrated HAstV replication induces EMT. To date, HAstV infection has not been associated as a risk factor for developing any type of cancer. This makes astrovirus unique as the majority of viruses known to induce EMT are oncogenic [49]. The data presented here not only provides increased knowledge on astrovirus pathogenesis but also induction of EMT from by a non-oncogenic virus. Future studies will examine the activation of TGF-β by astrovirus and if the EMT process contributes to disease *in vivo*.

## Materials and methods

### Cells and virus propagation

The human intestinal adenocarcinoma cell line Caco-2 was obtained from ATCC (HTB-37). Cells were propagated in minimum essential medium (MEM; Corning) supplemented with 20% fetal bovine serum (FBS; HyClone), GlutaMax-I (Gibco), and 1 mM sodium pyruvate (Gibco). The human colorectal adenocarcinoma cell line HT29 was obtained from ATCC (HTB-38). HT29 cells were propagated in Dulbecco’s Modified Eagle’s Medium (DMEM; Corning) supplemented with 10% fetal bovine serum (FBS; HyClone).

HAstV-1, HAstV-2, and HAstV-8 lab adapted viral stocks were propagated in Caco-2 cells. Infectious titers were quantitated on Caco-2 cells by the fluorescent-focus assay as previously described [50]. To UV inactivate the virus, 100μl of HAstV-1 was subjected to 100 mJ/cm^2^ with a UV cross-linker as described previously [15]. Inactivation was confirmed by the fluorescent-focus assay. Clinical isolates (SJ054.225, SJ60.212, and SJ177.110) were isolated as previously described [51].

The reovirus T1l and T3SA+ strains were generous gifts from Dr. Terence Dermody’s lab at the University of Pittsburgh. HAstV-VA1 was a gift from Dr. David Wang’s lab at Washington University in St. Louis.

### Immunofluorescent staining

Briefly, Caco-2 cells were seeded onto glass coverslips (for epithelial and vimentin staining) or transwells (polarity and SMAD3 staining). Once confluent, the cells were infected with HAstV-1 (MOI 10) or mock infected. At various times post-infection, cells were fixed with 4% paraformaldehyde (for epithelial and vimentin staining) or 100% ice cold methanol (polarity and SMAD3 staining), and then blocked with 5% normal goat serum (NGS) in PBS at room temperature for 1 hour. The cells were stained for E-cadherin (33–4000; Invitrogen), occludin (71–1500; Invitrogen), ZO-1 (33–9100 and 61–7300; Invitrogen), sodium-potassium ATPase (ab167390; abcam), ezrin (MA5-13862; Invitrogen), β-catenin (ab32572; abcam), SMAD3 (51–1500; Invitrogen), HAstV capsid (8e7; DakoCytomation), and vimentin (ab92547; abcam) for 1 hour followed by anti-mouse IgG-Alexa Fluor 488 or anti-rabbit IgG-Alexa Fluor 555 (Invitrogen) secondary antibodies and DAPI (4′,6′-diamidino-2-phenylindole; Sigma) in 1% NGS for 30 min at room temperature. Following staining, coverslips or transwells were mounted onto slides with Prolong Gold Antifade Mountant (Invitrogen) and sealed. Cells were imaged with a Nikon TE2000 inverted microscope Images were captured with a Nikon 60x objective lens using Nikon NIS Elements software.

### Western blotting

Caco-2 cells were mock- or HAstV-1 (MOI 10) infected or were treated with an equal amount of UV-inactivated virus. At the indicated times, monolayers were lysed in 100 μl of RIPA buffer (Abcam) and 1× protease inhibitor cocktail (Pierce) for 15 min at room temperature and centrifuged at 14,000 × *g* for 5 min at 4°C. Protein concentrations were determined using the BCA Protein Assay Kit (Pierce). Equal protein concentrations of the soluble fraction were separated by sodium dodecyl sulfate-polyacrylamide gel electrophoresis (SDS-PAGE) (4–20%) under reducing conditions. Following transfer to nitrocellulose and probing for for E-cadherin (33–4000; Invitrogen), occludin (71–1500; Invitrogen), ZO-1 (33–9100; Invitrogen), β-catenin (ab32572; abcam), vimentin (ab92547; abcam), and β-actin (A5441; Sigma). The blot was imaged on Licor Odyssey Fc and band densitometry was measured using Image Studio version 5.2 software.

### Gene expression analysis by microarray

Total RNA (100 ng) was converted to biotin-labeled cDNA using the Affymetrix WT Plus kit and hybridized to an Affymetrix Human Gene 2.0 ST GeneChip array (Life Technologies). Array probes were normalized and summarized to transcript-level signals by the RMA algorithm using the Affymetrix Expression Console software v1.1. Gene Set Enrichment Analysis (GSEA) was performed as described [52] using gene sets downloaded from MSigDB (https://www.gsea-msigdb.org/gsea/msigdb).

### RT^2^ profiler

Briefly, cells were seeded in 6-well plate, mock infected or HAstV-1 (MOI 10) infected and collected at the indicated time point in TRIzol reagent (Thermo Fisher Scientific). Then, RNA was isolated according to the manufacturer’s instructions. RNA quality was determined and was reverse transcribed using Qiagen’s RT^2^ First Strand Kit (Cat# 330401). The cDNA was used on the real-time RT^2^ Profiler PCR Array (Cat# PAHS-090Z) in combination with RT^2^ SYBR Green qPCR Mastermix (Cat# 330529). The CT values were then uploaded on to the data analysis web portal at http://www.qiagen.com/geneglobe. Samples were assigned to either control or test groups. The data was normalized based on a manual selection from full panel of reference genes. The data analysis web portal calculated fold change/regulation using ΔΔCT method, in which ΔCT is calculated between gene of interest and an average of reference genes (B2M, HPRT1, and RPLP0), followed by ΔΔCT calculations (ΔCT (Test Group)-ΔCT (Control Group)). Fold Change was then calculated using 2^ (-ΔΔCT) formula.

### RT-PCR

Caco-2 cells were infected with HAstV-1 or mock infected and RNA extracted at indicated timepoints using TRIzol (AMbion) according to manufacturer’s specifications. Then qRT-PCR was performed using the QuantiTect SYBR green kit (Qiagen) primer assays for *OCLN* (cat# QT00081844), *CDH1* (cat# QT00080143), *SERPINE1* (cat# QT00062496), *SNAI1* (cat# QT00010010), *TGFB1* (cat# QT00000728), *TWIST1* (cat# QT00011956), *ZEB1* (cat# QT00020972), *ZEB2* (cat# QT00008554), and *VIM1* (cat# QT00095795). The resulting Ct values were normalized to *GAPDH* (cat# QT00079247). The log transformed ΔΔCt values are reported as fold changes over untreated.

### Quantification of sodium potassium ATPase staining

Following immunofluorescent staining and imaging, basal or apical localization of sodium-potassium ATPase was determined by measuring mean fluorescent intensity above and below cell midline using ImageJ 1.50i software. Results were expressed as a ratio of basal fluorescent intensity to apical fluorescent intensity.

### TGF-β activity assay

TGF-β activity was measured using a luciferase reporter cell line, as previously described [34]. Briefly, mink lung epithelial cells (Mv1Lu), stably transfected with a luciferase reporter construct downstream of the plasminogen activator inhibitor-1 (PAI-1) promotor, were plated in a 96-well tissue culture plate (2 x 10^4^). These cells were inoculated with supernatants (100 μl) taken from HAstV-1 (MOI 10) or mock-infected Caco-2 cells at various times post-infection and incubated at 37°C for 16–20 hours. The inoculum was removed, and the cells washed twice with PBS. Cell lysates were prepared and assayed for luciferase activity using the Luciferase Assay System (Promega) and imaged on the Cytation 5 Cell Imaging Multi-Mode Reader (BioTek).

### SB431542 and U0126 treatment

Briefly, 5 × 10^4^ cells were seeded into transwells (3074; Corning), and once confluent, transferred into serum free media for at least 1 hour. The cells were treated with 10μM U0126 (Promega) or 20μM SB431542 (Tocris) 1 hour prior to infection. Then the cells were infected with HAstV-1 (MOI 5), TGF-β treated (20 ng/ml), or mock infected, according to experiment, in serum free media. Following the virus adsorption period of 1 hour, the inoculum was removed and fresh media containing 10μM U0126 or 20μM SB431542 was replaced. DMSO was used as the vehicle control in both experimental setups.

### Supernatant titering

Supernatants were titered via fluorescent focus assay as previously described [50]. Viral RNA was extracted from supernatants with QIAamp Viral RNA Mini Kit (Qiagen). Quantitative PCR (qPCR) was performed as previously described [53].

### Lactate dehydrogenase (LDH) assay

Cytotoxicity was measured using the LDH Assay Kit (abcam) per the manufacturer’s protocol. Briefly, at 8- and 24-hours post-infection, 100μl of supernatant was combined with 100μl of LDH Reaction Mixture and incubated for 30 min in the dark at room temperature. The absorbance of the samples at 490nm was read on a microtiter plate reader. The percent of cytotoxicity was calculated as a percent of the positive control (cells treated with 1% Triton X-100) normalized to mock-infected wells.

### Statistical analysis

Data were analyzed by ordinary one-way ANOVA followed by Dunnett’s multiple comparisons test (RT-PCR of epithelial and mesenchymal genes), ordinary one-way ANOVA followed by a test for trend (epithelial protein expression), two-tailed student t-test (Na/K-ATPase localization), two-way ANOVA followed by Sidak’s multiple comparisons test (TGF-β Activity, percent of HAstV-1 infected cells with and without SB431542 treatment, and supernatant titers quantified by FFU and genome copy), ordinary one-way ANOVA followed by Dunnett’s multiple comparisons test (E-cad expression with UV-inactivated virus, and U0126, and RT-PCR with UV-inactivated virus), and ordinary one-way ANOVA followed by Tukey’s multiple comparisons test (E-cad expression and RT-PCR with SB431542) using GraphPad Prism version 9. Asterisks show statistical significance as follows: *, P < 0.05; **, P < 0.01; ***, P < 0.001.

## Supporting information

S1 Fig EMT pathway is upregulated in HAstV-infected Caco-2 cells. Gene set enrichment analysis was performed on HAstV-infected (MOI of 10) and uninfected Caco-2 intestinal epithelial cells. Shown are top upregulated hallmark pathways’ normalized enrichment scores with false discovery rate estimated by Benjamin-Hochberg method cut-offs of q<0.001 (red), q<0.05 (blue), and q>0.05 or non-significant (gray).
(TIF)

S2 Fig EMT-associated genes modulated by HAstV infection. Heatmap showing fold regulation of EMT associated genes from Qiagen’s RT^2^ Profiler PCR Array Human Epithelial to Mesenchymal Transition (EMT). RNA samples were collected from HAstV-infected or mock-infected cells at 8 and 24 hpi. Gene expression values are colored corresponding to the up (red) or downregulation (blue) relative to mock-infected cells.
(TIF)

S3 Fig HAstV-1 infection does not lead to significant cell death within 24 hours. Cell toxicity was measured by lactate dehydrogenase (LDH) assay at 8- and 24-hours post-infection of HAstV-1, VA1, T1L, and T3SA (MOI 10). Error bars indicate standard deviations of two independent experiments performed in triplicate.
(TIF)

S4 Fig Multiple HAstV serotypes and clinical isolates induce EMT. (A) Caco-2 monolayers on grown coverslips, infected with HAstV-1 (lab-adapted), SJ054.225 (HAstV-1 isolate), HAstV-8 (lab-adapted), SJ60.212 (HAstV-8 isolate), HAstV-2 (lab-adapted), SJ177.110 (HAstV-2 isolate) or mock infected. Cells were fixed at 24 hpi and stained for E-cadherin (red) and DAPI (blue). (B) Expression of E-cadherin was quantified by immunoblot of HAstV-1 (lab adapted), SJ054.225 (HAstV-1 isolate), HAstV-8 (lab adapted), SJ60.212 (HAstV-8 isolate), HAstV-2 (lab adapted), SJ177.110 (HAstV-2 isolate) infected (MOI of 10) or mock infected Caco-2 cell lysates. Bands were then quantified by densitometry and normalized to β-actin then compared to mock-infection. Error bars indicate standard deviations of two independent experiments performed in triplicate, and asterisks show statistical significance as measured by ordinary one-way ANOVA followed by Dunnett’s multiple comparisons test as follows: *, P < 0.05; **, P < 0.01; ***, P < 0.001. (C) Na/K ATPase (red) and ezrin (green) localization in Caco-2 cells infected with lab adapted and clinical isolate HAstVs is disrupted compared to mock infected cells. All images are representative of two independent experiments.
(TIF)

S5 Fig HAstV-1 induces EMT in HT29 cells to a lesser degree than Caco-2 cells. (A) HT29 and Caco-2 monolayers infected with HAstV-1 were fixed at 24 hpi and stained for E-cadherin (red), HAstV capsid (green) and DAPI (blue). (B) Expression of E-cadherin was quantified by immunoblot of mock- and HAstV-1 infected (MOI 10) Caco-2 and HT29 cell lysates. Bands were then quantified by densitometry and normalized to β-actin then compared to mock-infection. Error bars indicate standard deviations of one experiment performed in duplicate. (C) *SERPINE1*, *SNAI1*, and *CDH1* mRNA levels were measured in mock and HAstV-1 infected (MOI 5) Caco-2 and HT29 cells at 24 hpi. Error bars indicate standard deviations from two independent experiments performed in duplicate, and asterisks show statistical significance as measured by ordinary one-way ANOVA followed by Tukey’s multiple comparisons test as follows: *, P < 0.05; ****, P < 0.0001.
(TIF)

S6 Fig TGF-β induced EMT in Caco-2 cells. (A) Western blot of E-cadherin at 1-, 2-, 3-, and 4-days post inoculation with 20 ng/ml active TGF-β compared to mock. Bands were quantified by densitometry and normalized to β-actin then compared to mock-infection. (B) Caco-2 inoculated with 20ng/ml active TGF-β or mock treated (as indicated). Cells were fixed at 1-, 2-, or 3-days post-inoculation in 100% ice-cold methanol and then stained for ezrin (green), Na/K ATPase (red), and DAPI (blue).
(TIF)
