# Supplementary figures and images for: Astrovirus-induced epithelial-mesenchymal transition via activated TGF-β increases viral replication
Source: PLoS Pathog. 2022 Apr 22;18(4):e1009716. doi: 10.1371/journal.ppat.1009716 (PMC9067694; doi:10.1371/journal.ppat.1009716)

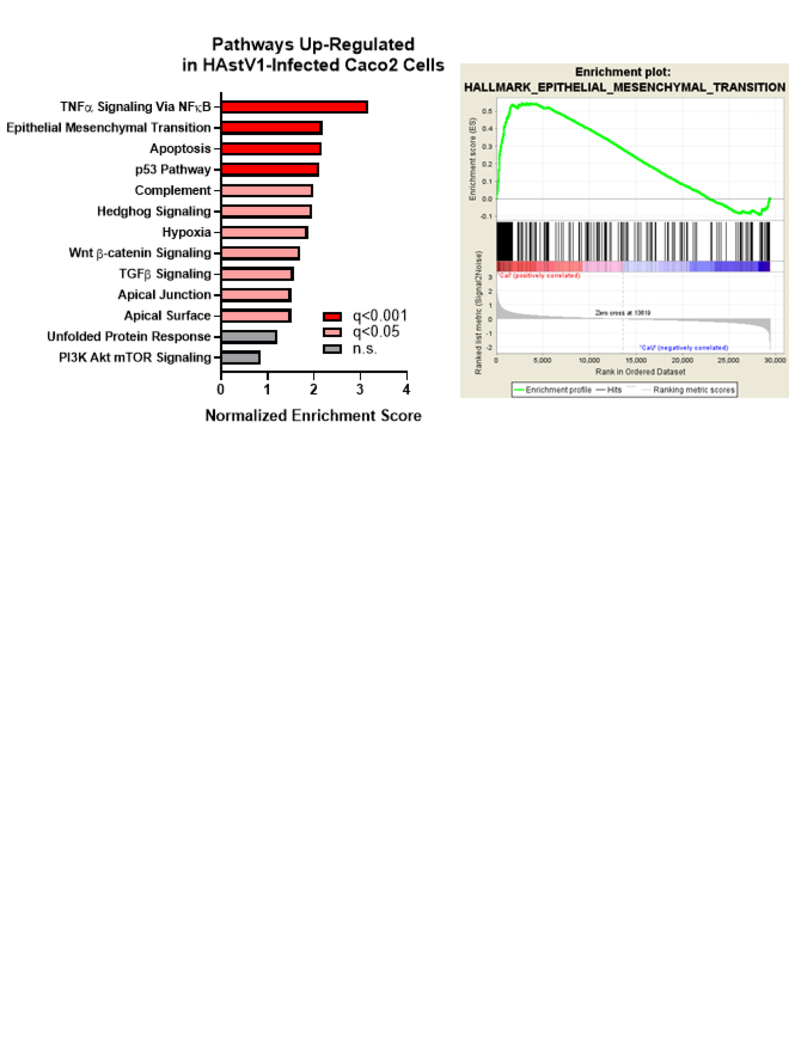

Supplement: S1 Fig — Gene set enrichment analysis was performed on HAstV-infected (MOI of 10) and uninfected Caco-2 intestinal epithelial cells. Shown are top upregulated hallmark pathways’ normalized enrichment scores with false discovery rate estimated by Benjamin-Hochberg method cut-offs of q<0.001 (red), q<0.05 (blue), and q>0.05 or non-significant (gray). (TIF) [file ppat.1009716.s001.tif]

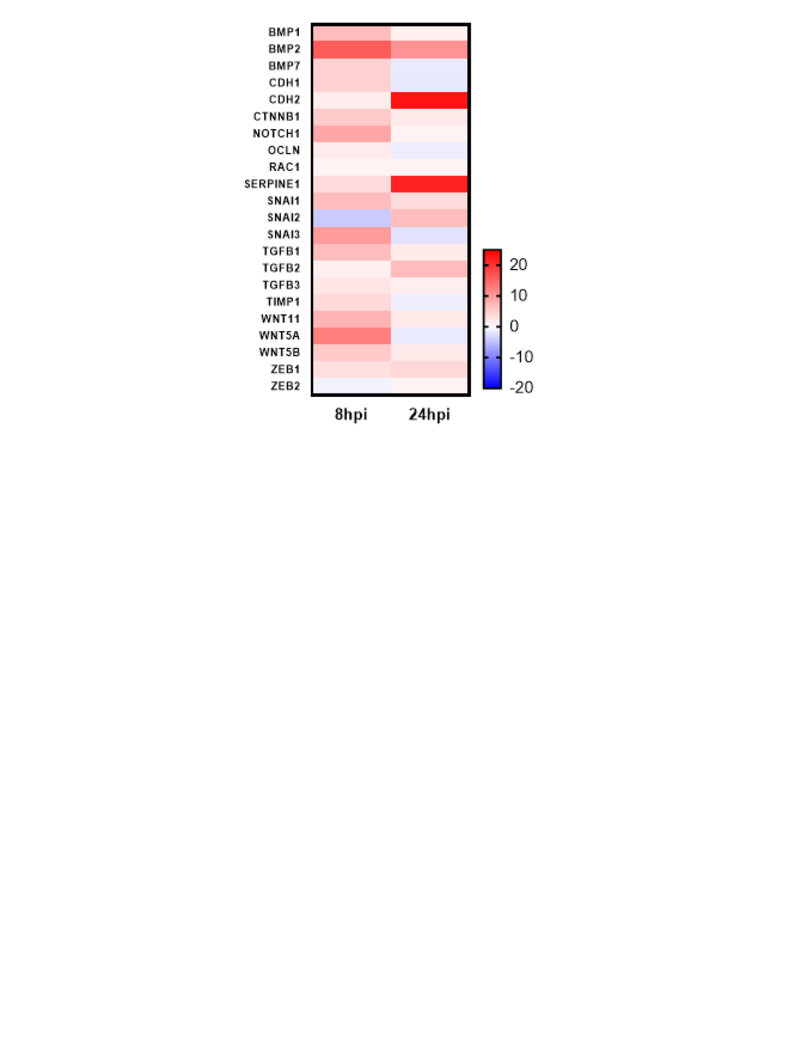

Supplement: S2 Fig — Heatmap showing fold regulation of EMT associated genes from Qiagen’s RT2 Profiler PCR Array Human Epithelial to Mesenchymal Transition (EMT). RNA samples were collected from HAstV-infected or mock-infected cells at 8 and 24 hpi. Gene expression values are colored corresponding to the up (red) or downregulation (blue) relative to mock-infected cells. (TIF) [file ppat.1009716.s002.tif]

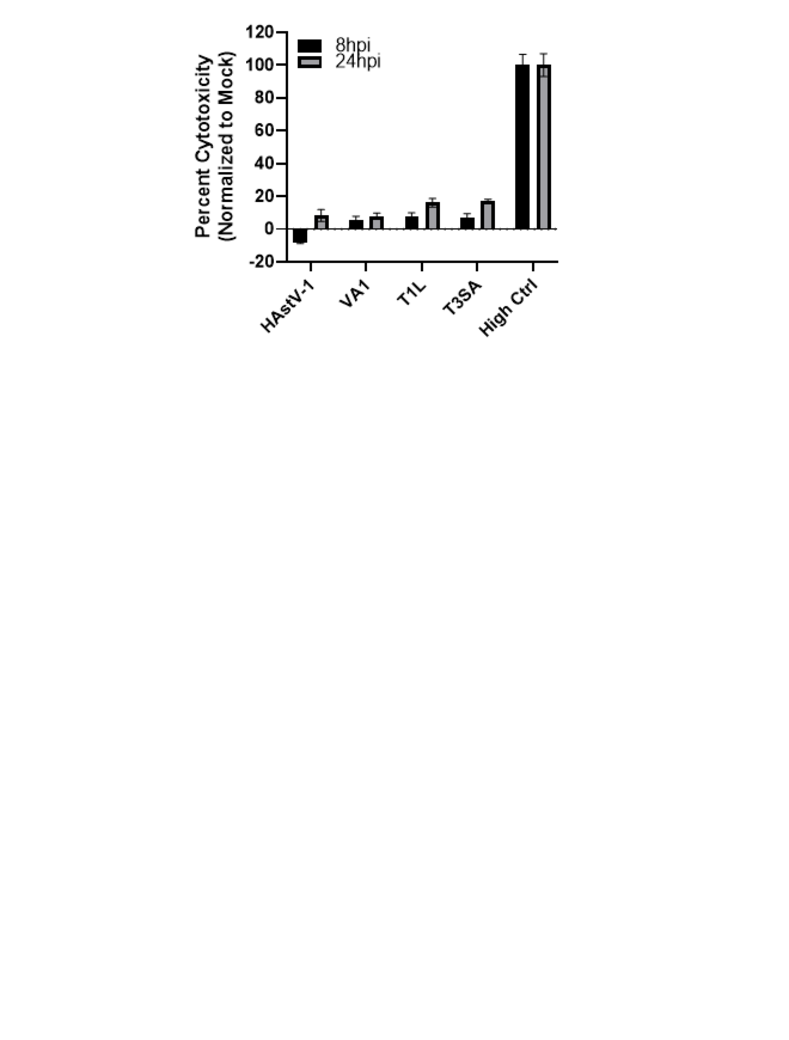

Supplement: S3 Fig — Cell toxicity was measured by lactate dehydrogenase (LDH) assay at 8- and 24-hours post-infection of HAstV-1, VA1, T1L, and T3SA (MOI 10). Error bars indicate standard deviations of two independent experiments performed in triplicate. (TIF) [file ppat.1009716.s003.tif]

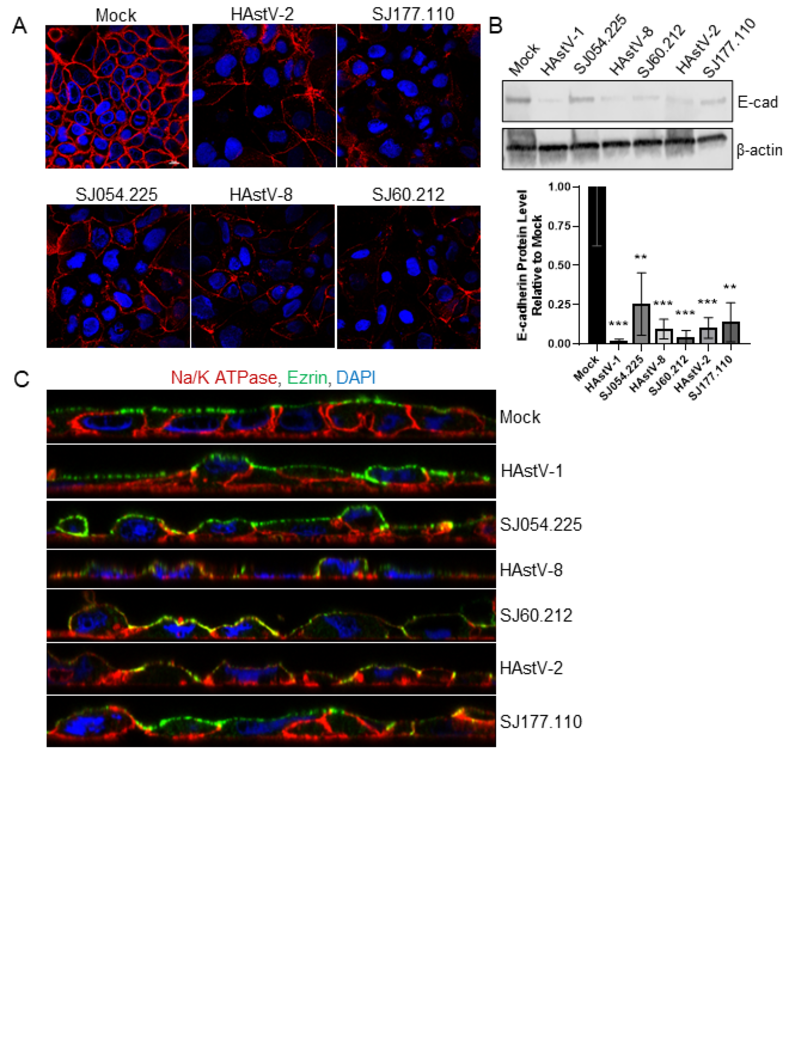

Supplement: S4 Fig — (A) Caco-2 monolayers on grown coverslips, infected with HAstV-1 (lab-adapted), SJ054.225 (HAstV-1 isolate), HAstV-8 (lab-adapted), SJ60.212 (HAstV-8 isolate), HAstV-2 (lab-adapted), SJ177.110 (HAstV-2 isolate) or mock infected. Cells were fixed at 24 hpi and stained for E-cadherin (red) and DAPI (blue). (B) Expression of E-cadherin was quantified by immunoblot of HAstV-1 (lab adapted), SJ054.225 (HAstV-1 isolate), HAstV-8 (lab adapted), SJ60.212 (HAstV-8 isolate), HAstV-2 (lab adapted), SJ177.110 (HAstV-2 isolate) infected (MOI of 10) or mock infected Caco-2 cell lysates. Bands were then quantified by densitometry and normalized to β-actin then compared to mock-infection. Error bars indicate standard deviations of two independent experiments performed in triplicate, and asterisks show statistical significance as measured by ordinary one-way ANOVA followed by Dunnett’s multiple comparisons test as follows: *, P < 0.05; **, P < 0.01; ***, P < 0.001. (C) Na/K ATPase (red) and ezrin (green) localization in Caco-2 cells infected with lab adapted and clinical isolate HAstVs is disrupted compared to mock infected cells. All images are representative of two independent experiments. (TIF) [file ppat.1009716.s004.tif]

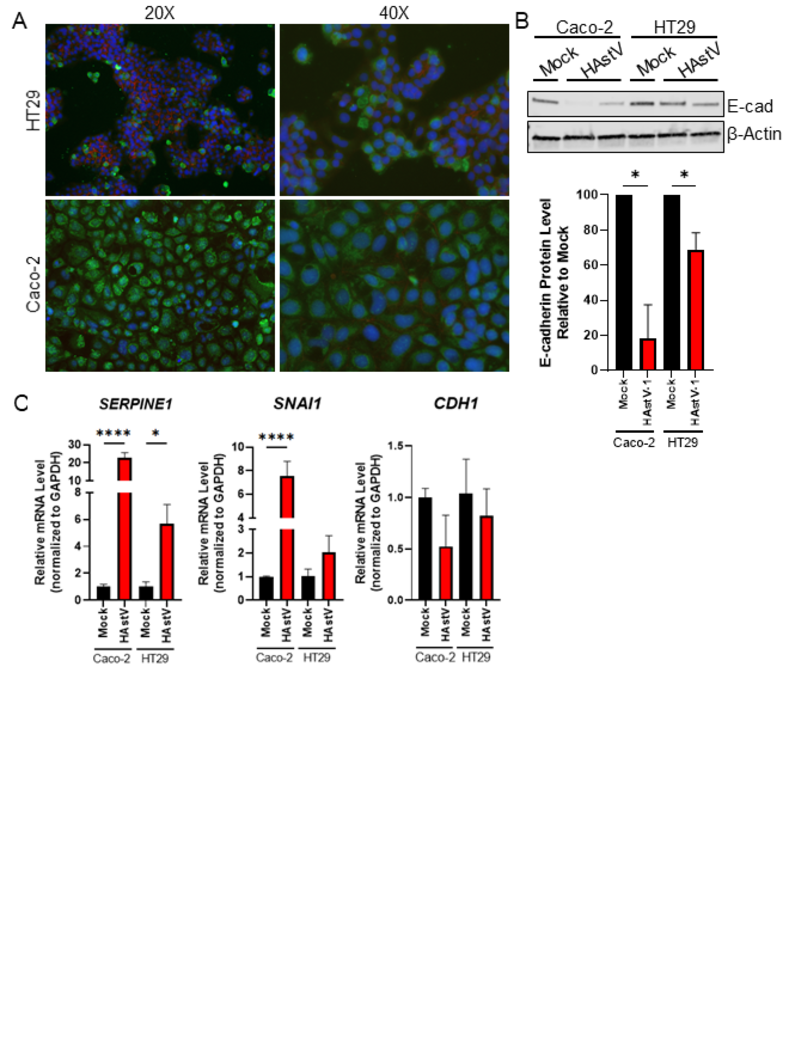

Supplement: S5 Fig — (A) HT29 and Caco-2 monolayers infected with HAstV-1 were fixed at 24 hpi and stained for E-cadherin (red), HAstV capsid (green) and DAPI (blue). (B) Expression of E-cadherin was quantified by immunoblot of mock- and HAstV-1 infected (MOI 10) Caco-2 and HT29 cell lysates. Bands were then quantified by densitometry and normalized to β-actin then compared to mock-infection. Error bars indicate standard deviations of one experiment performed in duplicate. (C) SERPINE1, SNAI1, and CDH1 mRNA levels were measured in mock and HAstV-1 infected (MOI 5) Caco-2 and HT29 cells at 24 hpi. Error bars indicate standard deviations from two independent experiments performed in duplicate, and asterisks show statistical significance as measured by ordinary one-way ANOVA followed by Tukey’s multiple comparisons test as follows: *, P < 0.05; ****, P < 0.0001. (TIF) [file ppat.1009716.s005.tif]

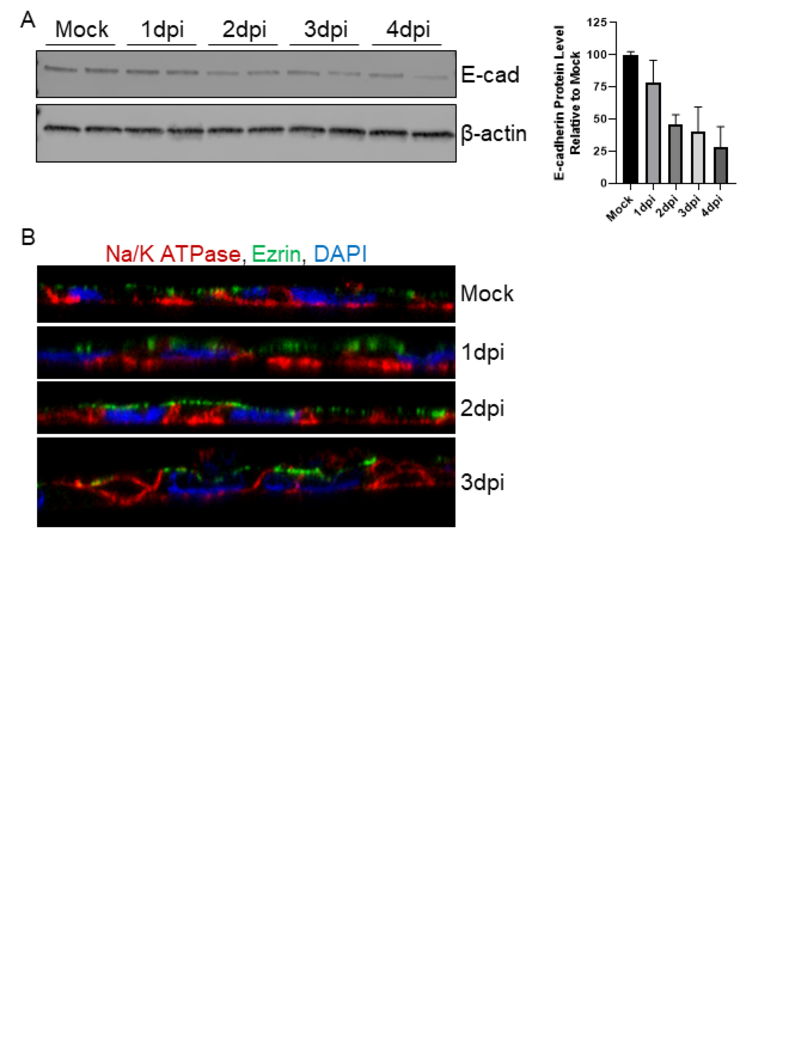

Supplement: S6 Fig — (A) Western blot of E-cadherin at 1-, 2-, 3-, and 4-days post inoculation with 20 ng/ml active TGF-β compared to mock. Bands were quantified by densitometry and normalized to β-actin then compared to mock-infection. (B) Caco-2 inoculated with 20ng/ml active TGF-β or mock treated (as indicated). Cells were fixed at 1-, 2-, or 3-days post-inoculation in 100% ice-cold methanol and then stained for ezrin (green), Na/K ATPase (red), and DAPI (blue). (TIF) [file ppat.1009716.s006.tif]
